# Drp-1 as Potential Therapeutic Target for Lipopolysaccharide-Induced Vascular Hyperpermeability

**DOI:** 10.1155/2020/5820245

**Published:** 2020-06-26

**Authors:** Xu Luo, Shumin Cai, Yunfeng Li, Guicheng Li, Yuanyuan Cao, Chenmu Ai, Youguang Gao, Tao Li

**Affiliations:** ^1^Department of Critical Care Medicine, The People's Hospital of Longhua, Shenzhen 518109, China; ^2^Department of Critical Care Medicine, Nanfang Hospital, Southern Medical University, Guangzhou 510515, China; ^3^Department of Critical Care Medicine, The First People's Hospital of Chenzhou, Affiliated Chenzhou Hospital, Southern Medical University, Chenzhou 423000, China; ^4^Department of Anesthesiology, The First Affiliated Hospital of Fujian Medical University, The First School of Clinical Medicine, Fujian Medical University, Fuzhou 350005, China

## Abstract

Mitochondria-dependent apoptotic signaling has a critical role in the pathogenesis of vascular hyperpermeability (VH). Dynamin-related protein-1- (Drp-1-) mediated mitochondrial fission plays an important role in mitochondrial homeostasis. In the present study, we studied the involvement of Drp-1 in resistance to VH induced by lipopolysaccharide (LPS). To establish the model of LPS-induced VH, LPS at 15 mg/kg was injected into rats *in vivo* and rat pulmonary microvascular endothelial cells were exposed to 500 ng/ml LPS *in vitro*. We found that depletion of Drp-1 remarkedly exacerbated the mitochondria-dependent apoptosis induced by LPS, as evidenced by reduced apoptosis, mitochondrial membrane potential (MMP) depolarization, and activation of caspase-3 and caspase-9. Increased FITC-dextran flux indicated endothelial barrier disruption. In addition, overexpression of Drp-1 prevented LPS-induced endothelial hyperpermeability and upregulated mitophagy, as evidenced by the loss of mitochondrial mass and increased PINK1 expression and mitochondrial Parkin. However, the mitophagy inhibitor, 3-Methyladenine, blocked these protective effects of Drp-1. Furthermore, inhibition of Drp-1 using mitochondrial division inhibitor 1 markedly inhibited LPS-induced mitophagy and aggravated LPS-induced VH, as shown by increased FITC-dextran extravasation. These findings implied that Drp-1 strengthens resistance to mitochondria-dependent apoptosis by regulating mitophagy, suggesting Drp-1 as a possible therapeutic target in LPS-induced VH.

## 1. Introduction

Sepsis is associated with the pathogenesis of multiorgan failure, and one of the important clinical features of sepsis is vascular hyperpermeability (VH), which results from disruption of the endothelial cell barrier, representing a challenging issue in critical care [1–3]. In recent years, animal models have identified many agents related to protection against VH; however, they all failed in clinical trials. Therefore, there is an urgent need to develop novel molecular targets that could be translated into effective VH therapies.

Our previous study reported that the pathogenesis of LPS-induced endothelial barrier disruption involved the mitochondria-mediated intrinsic apoptotic signaling pathway [1]. When mitochondria-dependent apoptotic signaling is activated, caspase-3 activation results in disruption of cell-cell adhesion in endothelial cells, ultimately leading to VH [4].

Considering the involvement of the mitochondria-dependent apoptotic signaling pathway in the pathogenesis of endothelial barrier disruption [1], it seems obvious that resistance of VH would depend on maintaining mitochondrial homeostasis, especially clearing the damaged mitochondria. Mitochondria are dynamic organelles that constantly cycle through fusion, fission, biogenesis, and selective degradation [5]. Mitochondria frequently change their shape and number to adapt to environmental variations. Dynamin-related protein-1 (Drp-1), a member of the dynamin family of GTPases, mainly regulates mitochondrial fission [6]. Drp-1 translocates from the cytosol to the outer membrane and binds to its receptors on the mitochondrial outer membrane (MOM), ultimately splitting mitochondrial tubules into fragments [7, 8]. It has been suggested that because of their small size, fragmented mitochondria tend to be enveloped by autophagosomes, implying that mitochondrial fission regulates mitophagy [9]. A healthy population of mitochondria is maintained via mitophagy-induced removal of dysfunctional mitochondria [10, 11]. Translocation of Drp-1 to the mitochondrial membrane is required to eliminate damaged mitochondria through mitophagy [12]. However, the role of Drp-1 in sepsis-induced VH is unknown. Therefore, the present study is aimed at determining the involvement of Drp-1 in the resistance to lipopolysaccharide- (LPS-) induced damage to the endothelial cell barrier.

## 2. Materials and Methods

### 2.1. Antibodies and Reagents

Molecular Probes (Invitrogen, CA, USA) provided MitoProbe™ JC-1 (5,5′,6,6′-tetrachloro-1,1′,3,3′-tetraethyl-imidacarbocyanine iodide), and MitoTracker Red, BestBio Co. (Beijing, China) provided the mitochondrial/cytosolic protein extraction kit. Antibodies recognizing translocase of outer mitochondrial membrane 20 (TOMM20), translocase of inner mitochondrial membrane 23 (TIMM23), PPARG coactivator 1 alpha (PGC-1*α*), transcription factor A mitochondria (TFAM), caspase-3, caspase-9, DRP-1, cytochrome C oxidase subunit 4 (COX4), tensin homolog (PTEN)-induced putative kinase 1 (PINK1), Parkin, beta-actin, and glyceraldehyde-3-phosphate dehydrogenase (GAPDH) were purchased from Abcam (Cambridge, UK). Keygen Biotech (Nanjing, China) provided the terminal deoxynucleotidyl transferase dUTP nick-end labeling (TUNEL) staining kit and the CellTiter-Glo assay. Promega Corp. (Madison, WI, USA) provided the luciferase-based assay kit. Guangzhou Cellcook Biotech Co., Ltd. (Guangzhou, China) provided the rat pulmonary microvascular endothelial cells (PMVECs). Sigma-Aldrich (Saint Louis, MO, USA) provided the fluorescein isothiocyanate- (FITC-) dextran and other chemicals.

### 2.2. Experimental Animals

The National Institutes of Health guidelines on the use of experimental animals were followed for the handling of the study animals and the animal procedures. The Ethics Committee of the First People's Hospital of Chenzhou approved the experimental protocol. The Experimental Animal Center at Southern Medical University provided the adult male Sprague-Dawley rats (weighing 180-220 g), which were permitted to acclimatize for 1 week prior to use. The rats had access to food and water *ad libitum*.

### 2.3. Cell Culture and Groups

Dulbecco's modified Eagle's medium (DMEM)/F12 containing FBS (10%) was used to culture PMVECs in a humidified atmosphere (95% air, 5% CO_2_) at 37°C. For the experiments, the PMVECs were grown to confluent cell monolayers on glass coverslips, microporous Transwell filter inserts, or culture dishes for 24 h. According to a previously described method [13], LPS at 500 ng/ml was used to induce endothelial barrier disruption for 24 h.

#### 2.3.1. The First Stage

PMVECs were allocated randomly into groups (*n* = 4): (1) control+vehicle (representing cells transfected with a scrambled small interfering RNA (siRNA) and exposed to normal conditions), (2) control+Drp-1-siRNA (cells transfected with an siRNA targeting *Drp-1* and exposed to normal conditions), (3) LPS+vehicle (cells transfected with a scrambled siRNA and exposed to LPS), and (4) LPS+Drp-1-siRNA (cells transfected with the siRNA targeting *Drp-1* and exposed to LPS).

#### 2.3.2. The Second Stage

PMVECs were allocated randomly into groups (*n* = 5): (1) control+vehicle (cells transfected with a null-plasmid and exposed to normal conditions), (2) control+Drp-1-plasmid (cells transfected with the Drp-1-plasmid and exposed to normal conditions), (3) LPS+vehicle (cells transfected with a null-plasmid and exposed to LPS), (4) LPS+Drp-1-plasmid (cells transfected with Drp-1-plasmid and exposed to LPS), and (5) LPS+3-Methyladenine (3MA)+Drp-1-plasmid (cells transfected with Drp-1-plasmid were treated with 3MA at 5 mM and exposed to LPS).

### 2.4. Dextran Transendothelial Flux

As described previously [13], a confluent monolayer of PMVECs was exposed to LPS in a Transwell chamber. FITC-dextran at 1 *μ*g/ml was added to the upper chamber and incubated for 45 minutes with the cells. Thereafter, an automatic microplate reader (SpectraMax M5; Molecular Devices, Sunnyvale, CA, USA) was used to determine and measure the concentration of the dextran in the upper and bottom chambers (excitation at 488 nm; emission at 525 nm).

### 2.5. Vascular Permeability Assay

Intramuscular injection of sodium pentobarbital (30 mg/kg) was used to anesthetize the rats. LPS (5 mg/ml) was injected into the tail vein to establish the model of LPS-induced vascular hyperpermeability [13]. The method of Shumin (2018) was used to measure vascular permeability. In brief, rats were positioned on the platform of an intravital upright microscope (ECLIPSEFN1; Nikon, Tokyo, Japan). We then performed a midline laparotomy, and a portion of the mesentery from the proximal ileum was moved onto the optical stage and observed at 100x magnification. FITC-dextran (50 mg/kg) was then injected intravenously, and the postcapillary venules (diameter = 20–50 *μ*m) were observed using the intravital microscope. The following formula was used to calculate the change in permeability Δ*I* = *I*_o_/*I*_i_, where *I*_o_ is the light intensity outside the vessel and *I*_i_ is the light intensity inside the vessel. After the addition of LPS, the parameters were recorded at 0 and 60 min.

Rats were randomly divided into four groups (*n* = 6 per group): (1) control+vehicle (rats treated with vehicle (dimethyl sulfoxide (DMSO))), (2) control+mdivi-1 (rats treated with mitochondrial division inhibitor 1 (mdivi-1) (3 mg/kg) [10]), (3) LPS+vehicle group (rats that received LPS injection and followed by vehicle treatment), and (4) LPS+mdivi-1 group (rats that received LPS injection and followed by mdivi-1 (3 mg/kg) treatment).

### 2.6. siRNA and Plasmid Transfection

Santa Cruz Biotechnology (Santa Cruz, CA, USA) provided siRNA targeting *Drp-1*. The control is comprised of a scrambled nontargeting siRNA. Transfection of cells with the siRNAs was carried out following the supplier's instructions. In brief, exponentially growing cells were placed in the wells of six-well tissue culture plates (1 × 10^5^ cells per well) and incubated for 24 h. Oligofectamine and OPTI-MEMI-reduced serum medium were then used to mediate siRNA transfection of the cells.

To induce the overexpression of Drp-1, the mCh-Drp-1 plasmid was purchased from Addgene (Cambridge, UK). Lipofectamine 2000 was used to transfect 293T cells with mCh-Drp-1. Forty-eight hours after transfection, the cell supernatant was collected. This supernatant, containing adenovirus-Drp-1, was transfected into endothelial cells. Western blotting was used to identify cells stably expressing Drp-1. Transfection with the empty plasmid was used as a control. All experiments and treatments were performed after 48 h of cell transfection.

### 2.7. Mitochondrial Membrane Potential (MMP) and Mitochondrial Morphology Assays

The potential-sensitive fluorescent dye JC-1 was used to determine the MMP. LPS-treated cells and controls were incubated with JC-1 (5 *μ*mol/l) at 37°C for 15 min. JC-1 fluorescence in the cells was observed under an inverted fluorescent microscope (Ti-E Live Cell Imaging System, Nikon).

To visualize the mitochondria, cells were stained with 200 nM MitoTracker Red for 30 min and observed by a confocal microscope (LSM780; Zeiss Microsystems, Jena, Germany).

### 2.8. Cellular ATP Measurement

A luciferase-based assay (Promega) was used to determine intracellular ATP following the supplier's protocol. LPS-treated cells (100 *μ*l; 10,000 cells) were added with 100 *μ*l of reagent in the wells of a 96-well plate and incubated for 10 min at room temperature. An automatic microplate reader (SpectraMax M5; Molecular Devices, Sunnyvale, CA, USA) was used to record the generated luminescence.

### 2.9. TUNEL Staining

Cell apoptosis was assessed using TUNEL staining. The Hoechst reagent was used to stain the nuclei. Under 100x magnification, the emission of green fluorescence indicated apoptotic cells. The mean value of TUNEL-positive cells in 10 random visual fields was used to obtain the apoptotic index.

### 2.10. Western Blotting

Total proteins were obtained using lysis and homogenization from PMVECs and mesenteric microvasculature samples. A mitochondrial/cytosolic protein extraction kit (BestBio Co.) was then used to obtain cytoplasmic and mitochondrial proteins following the supplier's protocol.

The extracted total, mitochondrial, and cytoplasmic proteins were mixed with loading buffer, separated using SDS polyacrylamide gel electrophoresis with 10–15% acrylamide gels, and then transferred onto polyvinylidene fluoride membranes. The membranes were incubated with primary antibodies recognizing TOMM20, TIMM23, PGC-1*α*, TFAM, caspase-3, caspase-9, Drp-1, COX4, PINK1, Parkin, *ꞵ*-actin, and GAPDH; washed; and reacted with the appropriate secondary antibodies, and a chemiluminescence detection reagent was used to visualize the immunoreactive protein bands.

### 2.11. Statistical Analysis

The mean ± SD is used to represent all data. One-way analysis of variance (ANOVA) incorporating the least significant difference (LSD) multiple comparison test and Student's *t*-test, when appropriate, were used to determine the differences between groups. Statistical significance was accepted at *P* < 0.05.

## 3. Results

### 3.1. Inhibition of Drp-1 Exacerbates LPS-Induced Mitochondria-Dependent Apoptosis and Disruption of the Endothelial Barrier

**Figure 1:**
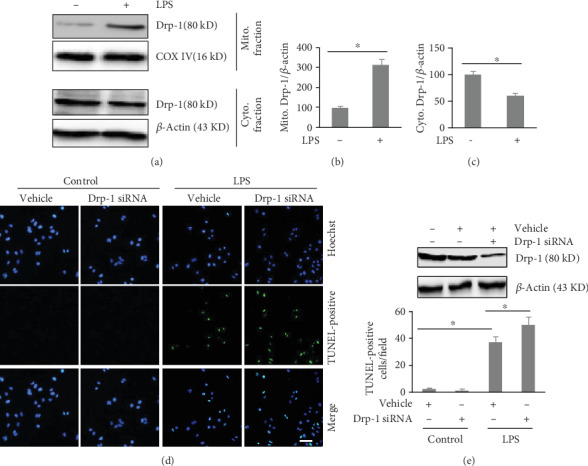
Depletion of Drp-1 exacerbates LPS-induced cell apoptosis. PMVECs were transfected with Drp-1 siRNA (a scrambled siRNA was used as the control) and exposed to LPS (500 ng/ml) for 24 h. (a) Western blotting evaluation of Drp-1 levels in cytosolic and mitochondrial cell fractions. (b) Mitochondrial Drp-1 level quantification; (c) cytoplasmic Drp-1 level quantification; (d) cellular apoptosis was assessed using TUNEL staining. Scale bars, 50 *μ*m. (e) The number of TUNEL-positive cells per field. The mean ± SD represent the data (*n* = 6 in each group). An asterisk (∗) indicates *P* < 0.05*vs*. the various groups. Drp-1: Dynamin-related protein-1; LPS: lipopolysaccharide; 3MA: 3-Methyladenine; TUNEL: terminal deoxynucleotidyl transferase nick-end labeling; COX IV: cytochrome C oxidase subunit 4.

Drp-1 regulates mitochondrial fission through translocating from the cytosol to the mitochondria and binding to its receptors. In the present study, we separately detected Drp-1 in the cytoplasm and mitochondria. The level of Drp-1 was increased in mitochondria and decreased in cytoplasm following LPS exposure, indicating the translocation of Drp-1 (Figures 1(a)–1(c)). *Drp-1* expression was then successfully silenced using an siRNA to determine whether Drp-1 plays a role in LPS susceptibility. We found that apoptosis was significantly increased in cells exposed to LPS, and this effect of LPS was exacerbated in cells transfected with the *Drp-1* siRNA (Figures 1(d) and 1(e)).

**Figure 2:**
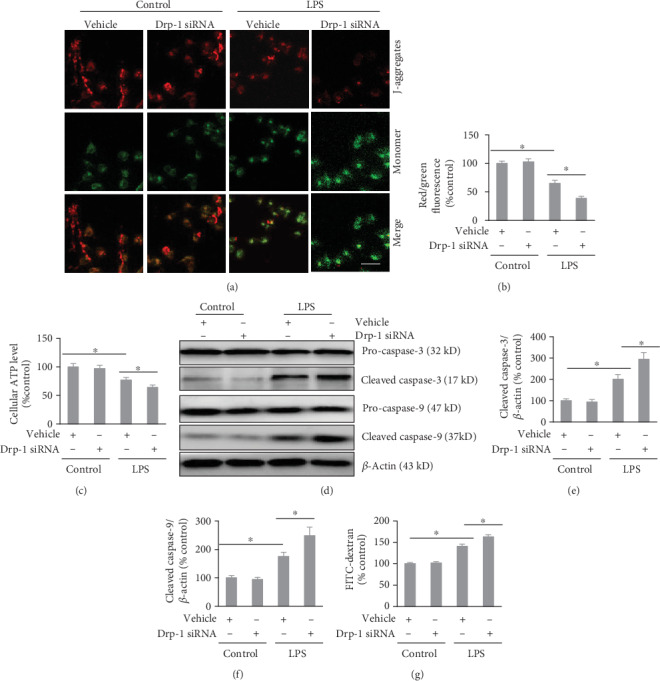
Depletion of Drp-1 aggravates LPS-induced activation of mitochondria-dependent apoptosis and endothelial barrier disruption. PMVECs were transfected with Drp-1 siRNA (a scrambled siRNA was used as the control) and exposed to LPS (500 ng/ml) for 24 h. (a) Fluorescent inverted microscopy (200x magnification) determination of JC-1 intracellular green and red fluorescence. Scale bars, 50 *μ*m. (b) JC-1 emitted intracellular green and red fluorescence quantification. (c) Luciferase-based assay for intracellular ATP levels. (d) Western blotting assessment of cleaved caspase-3 and cleaved caspase-9 levels. (e) Densitometry to quantify cleaved caspase-3 levels. (f) Densitometry to quantify cleaved caspase-9 levels. (g) FITC-dextran assessment of the permeability of an endothelial cell monolayer. The mean ± SD represent the data (*n* = 6 in each group). An asterisk (∗) indicates *P* < 0.05*vs*. the various groups. Drp-1: Dynamin-related protein-1; LPS: lipopolysaccharide; FITC: fluorescein isothiocyanate.

Previously, we demonstrated that mitochondria-dependent apoptosis has an important role in LPS-induced VH [10]. To investigate whether resistance to LPS-induced VH involves Drp-1, mitochondria-dependent apoptotic signaling and monolayer permeability were evaluated. The depolarized MMP, as demonstrated by increased JC-1 green and decreased JC-1 red fluorescence, decreased intracellular ATP levels, and increased levels of cleaved caspase-3 and cleaved caspase-9 were observed in LPS-treated cells, indicating activation of mitochondria-dependent apoptosis. Interestingly, lower MMP and intracellular ATP and higher cleaved caspase-3 levels and cleaved caspase-9 were detected in the LPS+Drp-1 siRNA group compared with those in the LPS+vehicle group (Figures 2(a)–2(f)). This indicated that Drp-1 depletion exacerbated LPS-induced activation of mitochondria-dependent apoptotic signaling. Furthermore, increased FITC-dextran flux was observed in the LPS+Drp-1 siRNA group compared with that in the LPS+vehicle group, which indicated that Drp-1 depletion exacerbated LPS-induced disruption of the endothelial barrier (Figure 2(g)).

### 3.2. Overexpression of Drp-1 Upregulates Mitophagy and Alleviates LPS-Induced Endothelial Hyperpermeability

**Figure 3:**
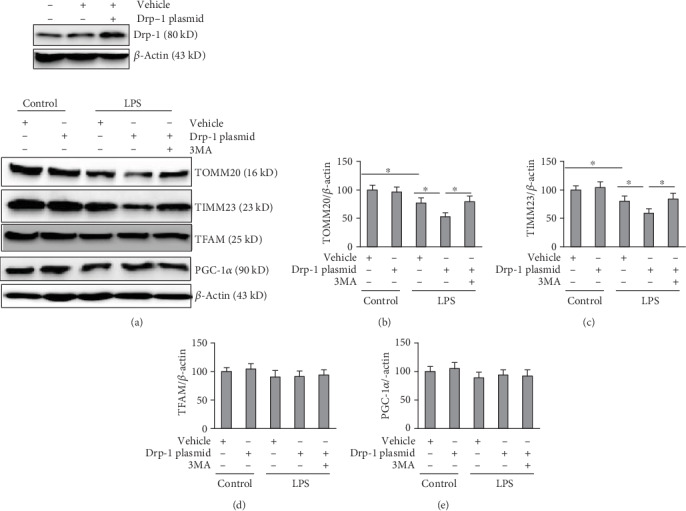
Overexpression of Drp-1 upregulates LPS-induced mitophagy. PMVECs transfected with Drp-1 plasmid (a null-plasmid was used as the control) were exposed to LPS (500 ng/ml) and treated with 3MA (5 mM) or a vehicle for 24 h. (a) Western blotting assessment of TOMM20, TIMM23, PGC-1*α*, and TFAM levels; (b) TOMM20 level quantification; (c) TIMM23 level quantification; (d) TFAM level quantification; (e) PGC-1*α* level quantification. The mean ± SD represent the data (*n* = 3 in each group). An asterisk (∗) indicates *P* < 0.05*vs*. the various groups. Drp-1: Dynamin-related protein-1; LPS: lipopolysaccharide; TOMM20: translocase of outer mitochondrial membrane 20; TIMM23: translocase of inner mitochondrial membrane 23; PGC-1*α*: PPARG coactivator 1 alpha; TFAM: transcription factor A mitochondria.

Mitophagy regulation involves an important contribution by Drp-1. In the present study, we detected the levels of TOMM20 and TIMM23, which are mitochondrial markers widely used to indicate mitophagy activity [10, 14]. The results showed that TOMM20 and TIMM23 levels were lower in the LPS+vehicle group compared with that in the control+vehicle group, indicating LPS-induced activation of mitophagy. Interestingly, decreased expression of TOMM20 and TIMM23 was detected in the LPS+Drp-1 plasmid group compared with that in the LPS+vehicle group, suggesting that overexpression of Drp-1 upregulated LPS-induced mitophagy (Figures 3(a)–3(c)). Next, we observed that Drp-1-induced mitophagy was inhibited by 3MA, an inhibitor of mitophagy. In addition, we determined the expression levels of mitochondrial biogenesis marker proteins PGC-1*α* and TFAM to eliminate the effects of mitochondrial biogenesis on the loss of mitochondrial mass. The results showed that in all groups, the levels of PGC-1*α* and TFAM did not change significantly (Figures 3(a), 3(d), and 3(e)).

**Figure 4:**
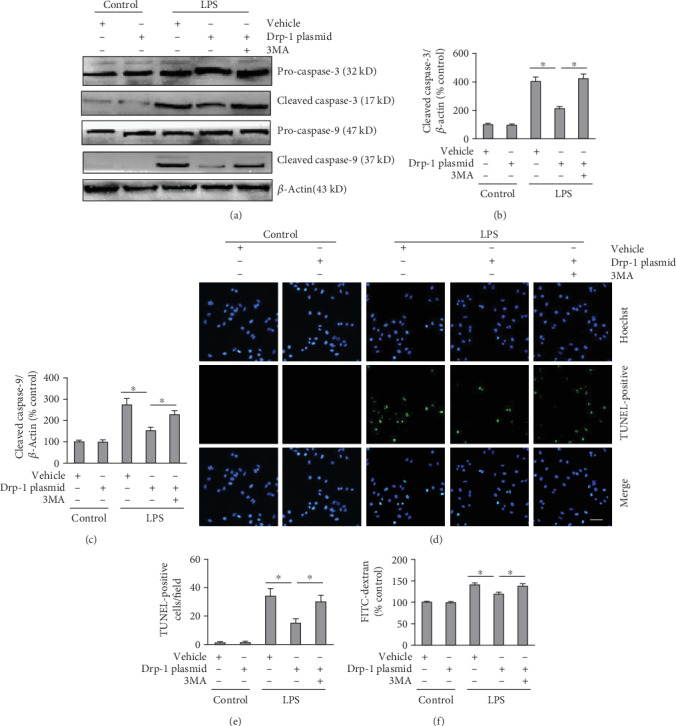
Overexpression of Drp-1 prevented LPS-induced endothelial hyperpermeability, which was blocked by 3MA. PMVECs transfected with Drp-1 plasmid (a null-plasmid was used as the control) were exposed to LPS (500 ng/ml) and treated with 3MA (5 mM) or a vehicle for 24 h. (a) Cleaved caspase-3 and cleaved caspase-9 levels were measured using Western blotting. (b) Quantification of cleaved caspase-3 expression using densitometry. (c) Quantification of cleaved caspase-9 expression using densitometry. (d) Apoptosis in cells was assessed using TUNEL staining. Scale bars, 50 *μ*m. (e) The number of TUNEL-positive cells per field. (f) FITC-dextran assessment of the permeability of an endothelial cell monolayer. The mean ± SD represent the data (*n* = 6 in each group). An asterisk (∗) indicates *P* < 0.05*vs*. the various groups. Drp-1: Dynamin-related protein-1; LPS: lipopolysaccharide; 3MA: 3-Methyladenine; FITC: fluorescein isothiocyanate; TUNEL: terminal deoxynucleotidyl transferase nick-end labeling.

Next, we confirmed that LPS-induced VH involved Drp-1. Overexpression of Drp-1 significantly prevented LPS-induced MMP depolarization, cellular ATP decrease, activation of caspase-3 and caspase-9, cell apoptosis, and FITC-dextran flux (Figure 4 and Supplementary Figure S2). These results suggested that upregulation of Drp-1 prevented LPS-induced activation of endothelial hyperpermeability and mitochondria-dependent apoptosis. Interestingly, these Drp-1-induced protective effects were inhibited by 3MA, suggesting that Drp-1 mediates LPS-induced mitochondria-dependent apoptosis and endothelial hyperpermeability via mitophagy (Figure 4 and Supplementary Figure S2).

### 3.3. Drp-1 Regulates Mitochondrial Fission and PINK1-Parkin-Mediated Mitophagy

It has been reported that Drp-1 mediated mitophagy by regulating mitochondrial fission; the mitochondrial morphology was detected in the present study. The mitochondria from normal cells display interconnected and filamentous shape; however, the mitochondria from cells in the LPS+vehicle group showed punctate and disconnected shape, indicating the mitochondrial fission (Supplementary Figure S1a). Interestingly, overexpression of Drp-1 facilitated LPS-induced mitochondrial fission, and inhibition of Drp-1 blocked LPS-induced mitochondrial fission (Supplementary Figure S1a).

PINK1-Parkin signaling is a key pathway involved in the regulation of mitophagy; we detected which to confirm the effects of Drp-1 on mitophagy. We found that overexpression of Drp-1 significantly increased the expression of PINK1 and the translocation of Parkin to mitochondria from cytosol as compared to the LPS+vehicle group, suggesting the upregulation of PINK1-Parkin-mediated mitophagy (Supplementary Figure S1b-e). Furthermore, inhibition of Drp-1 markedly blocked LPS-induced PINK1-Parkin-mediated mitophagy (Supplementary Figure S1b-e).

### 3.4. Inhibition of Drp-1 Blocked Mitophagy and Exacerbated LPS-Induced Vascular Hyperpermeability in Rats

**Figure 5:**
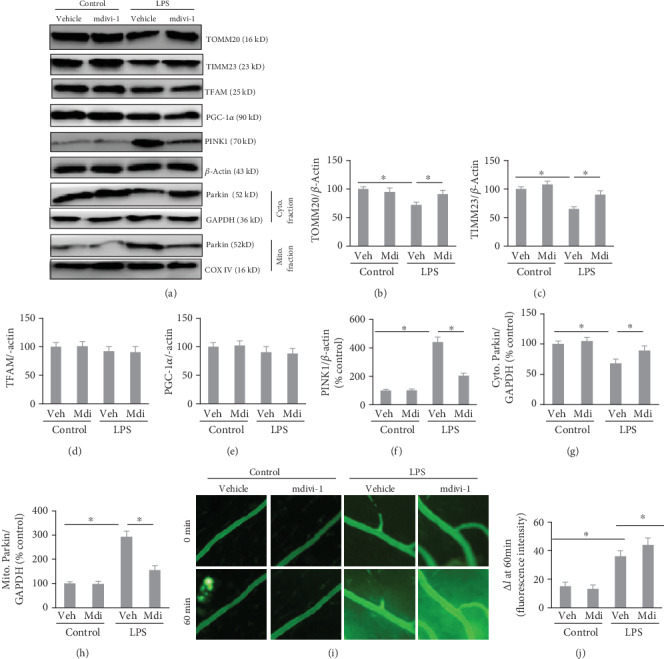
Inhibition of Drp-1 blocked mitophagy and exacerbated LPS-induced vascular hyperpermeability. Mice were subjected to LPS (5 mg/kg) and treated with either mdivi-1 (3 mg/kg) or vehicle for 60 min. (a) Western blotting was used to assess mitophagy-related proteins. (b) TOMM20 level quantification; (c) TIMM23 level quantification; (d) TFAM level quantification; (e) PGC-1*α* level quantification. (f) PINK1 level quantification; (g) cytoplasmic Parkin level quantification; (h) mitochondrial Parkin level quantification. (i) Intravital microscopy analysis (100x magnification) of mesenteric postcapillary venules with altered permeability (100x magnification). Scale bars, 50 *μ*m. (j) Quantification of vascular permeability. The mean ± SD represent the data (*n* = 6 in each group). An asterisk (∗) indicates *P* < 0.05*vs*. various groups. Drp-1: Dynamin-related protein-1; LPS: lipopolysaccharide; TOMM20: translocase of outer mitochondrial membrane 20; TIMM23: translocase of inner mitochondrial membrane 23; PGC-1*α*: PPARG coactivator 1 alpha; TFAM: transcription factor A mitochondria; PINK1: tensin homolog (PTEN)-induced putative kinase 1; Cyto.: cytoplasmic; Mito.: mitochondrial; COX IV: cytochrome C oxidase subunit 4.

To confirm the role of Drp-1 in LPS-induced mitophagy and vascular hyperpermeability, the pharmacological inhibitor of Drp-1, mdivi-1 [15], was used to block Drp-1 activity. Treatment with mdivi-1 ameliorated the LPS-induced downregulation of TOMM20 and TIMM23 in the mesenteric microvasculature, with no influence on mitochondrial biogenesis, and inhibited LPS-induced upregulation of PINK1 expression and translocation of Parkin to mitochondria; these data indicated blockade of mitophagy (Figures 5(a)–5(h)).

We then evaluated the alterations in vascular permeability via changes in FITC-dextran extravasation in rats. In the rats, a substantial increase in FITC-dextran extravasation into the extravascular space indicated LPS-induced VH. Interestingly, increased FITC-dextran extravasation was displayed in LPS-challenged rats that received mdivi-1 treatment compared with those without mdivi-1 treatment, indicating that blockade of Drp-1 exacerbated LPS-induced VH (Figures 5(i) and 5(j)).

## 4. Discussion

In our previous studies, we reported the important role of the mitochondria-dependent apoptotic signaling pathway in VH [1, 16]. Mitochondrial dysfunction, characterized by MMP depolarization and mitochondrial permeability transition pore opening, causes the release of second mitochondria-derived activator of caspase and cytochrome C into cytoplasm, which activates caspase-3. Then, cleavage of *β*-catenin by the activated caspase-3 regulates cell-cell adhesion in endothelial cells mediated by VE-cadherin, ultimately causing microvascular hyperpermeability. Therefore, maintaining mitochondrial homeostasis is crucial to the therapy for VH [4, 16].

Mounting evidence highlights that the dynamics of mitochondrial fusion/fission are important in mitochondrial homeostasis [17, 18]. Drp-1, a member of the dynamin family of GTPases, is the core regulatory molecule of mitochondrial fission. Translocation of Drp-1 from the cytoplasm into the mitochondrial outer membrane results in mitochondrial fission [19]. Although mitochondrial fusion is considered beneficial to maintaining mitochondrial morphology and mitochondrial function [8], studies have implied that appropriate mitochondrial fission seems to be a key cytoprotective mechanism that promotes clearance of damaged mitochondria to adapt to various stress states [5, 19, 20]. Zuo et al. reported that the resistance of hippocampal CA3 neurons affected by ischemia is reinforced by Drp-1, suggesting Drp-1 as a therapeutic target to treat brain ischemic stroke. It has been reported that inhibition of mitochondrial fission induced by mdivi-1 enhanced cisplatin-induced cytotoxicity, MMP loss, and reactive oxygen species (ROS) formation in cholangiocarcinoma cells [21]. Moore et al. (2019) reported that Drp-1 deficiency reduced muscle endurance and altered muscle adaptations in response to exercise training. However, the role of Drp-1 in maintaining the endothelial barrier is still largely unknown. The results of the present study showed that Drp-1 inhibition significantly aggravated LPS-induced mitochondria-dependent apoptotic signaling and VH, both *in vitro* and *in vivo*. In addition, Drp-1 upregulation alleviated LPS-induced apoptosis and endothelial barrier collapse.

Many studies have indicated that mitochondrial dynamics play an important role in regulating mitophagy [22–24]. Mitochondrial fission is a response to multiple stimulations and age-related diseases. When fragmented mitochondria regain their membrane potential, they can fuse back to a mitochondria network. However, if the damaged mitochondria fail to do so, they can be eliminated via mitophagy [9]. Some studies have suggested that decreased mitophagy results from loss of Drp-1-mediated fission and that overexpression of the Drp-1 results in mitophagy [19, 25, 26]. In the present study, we found that overexpression of Drp-1 facilitated mitochondrial fission and upregulated LPS-induced mitophagy, and the inhibitor of Drp-1, mdivi-1, significantly prevented LPS-induced mitophagy. Our finding suggested that Drp-1 positively regulates mitophagy, in which mitochondrial fission might play a significant role. The elimination of damaged mitochondria and the preservation of healthy mitochondrial population are mediated by mitophagy [10, 27]. Studies have demonstrated that Drp-1 exerts its physiological function by mediating mitophagy [28]. Li et al. reported that Drp-1 protected the renal tubular epithelial cell against unilateral ureteral obstruction by regulating PARK2-dependent mitophagy [29]. Zuo et al. reported that Drp-1 mediates mitophagy via mitochondrial fission to protect against mitochondrial dysfunction in cerebral ischemia [30]. In the present study, our data indicated that inhibition of mitophagy by 3MA significantly reversed Drp-1's protective effects against LPS-mediated mitochondria-dependent apoptosis and endothelial hyperpermeability.

## 5. Conclusions

In conclusion, the results presented here provide clear evidence that Drp-1 mediates protection against LPS-induced mitochondria-dependent apoptotic signaling and VH. Furthermore, we speculated that Drp-1 protects against VH by mediating LPS-induced mitophagy and hence the rapid removal of damaged mitochondria. Taken together, our findings propose that Drp-1 could be a potential target to develop therapeutic strategies to treat LPS-induced VH.

## Data Availability

The datasets used and/or analysed in this study will be made available by the corresponding author (Tao Li) upon reasonable request.

## Supplementary Materials

Supplementary Materials Supplementary Figure S1: Drp-1 regulates mitochondrial fission, and PINK1-Parkin-mediated mitophagy PMVECs were transfected with either Drp-1 siRNA or Drp-1 plasmid and exposed to LPS (500 ng/ml) for 24 h. (a) Mitochondria were stained by MitoTracker Red and imaged by laser scanning confocal microscopy. Scale bars, 10 *μ*m. (b) Western blotting was used to assess PINK1 and Parkin levels. (c) PINK1 level quantification; (d) cytoplasmic Parkin level quantification; (e) mitochondrial Parkin level quantification. The mean ± SD represent the data (*n* = 6 in each group). An asterisk (∗) indicates *P* < 0.05 vs. the various groups. Drp-1: Dynamin-related protein-1; LPS: lipopolysaccharide; PINK1: tensin homolog (PTEN)-induced putative kinase 1; Cyto.: cytoplasmic; Mito.: mitochondrial; COX IV: cytochrome C oxidase subunit 4. Supplementary Figure S2: PMVECs transfected with Drp-1 plasmid (a null-plasmid was used as the control) were exposed to LPS (500 ng/ml) and treated with 3MA (5 mM) or a vehicle for 24 h. (a) Fluorescent inverted microscopy (200x magnification) determination of JC-1 intracellular green and red fluorescence. Scale bars, 50 *μ*m. (b) JC-1 emitted intracellular green and red fluorescence quantification. (c) Luciferase-based assay for intracellular ATP levels. The mean ± SD represent the data (*n* = 6 in each group). An asterisk (∗) indicates *P* < 0.05 vs. the various groups. Drp-1: Dynamin-related protein-1; LPS: lipopolysaccharide.
