# Supplementary figures and images for: Drp-1 as Potential Therapeutic Target for Lipopolysaccharide-Induced Vascular Hyperpermeability
Source: Oxid Med Cell Longev. 2020 Jun 26;2020:5820245. doi: 10.1155/2020/5820245 (PMC7336239; doi:10.1155/2020/5820245)

Figure s1

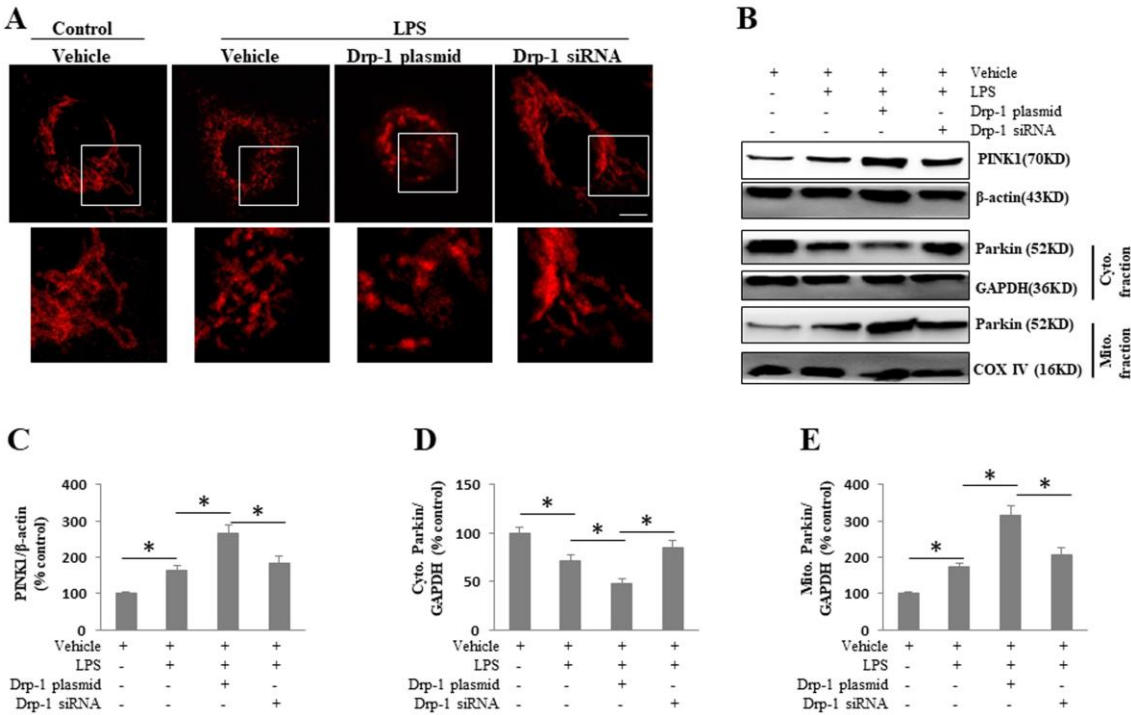

Figure s2

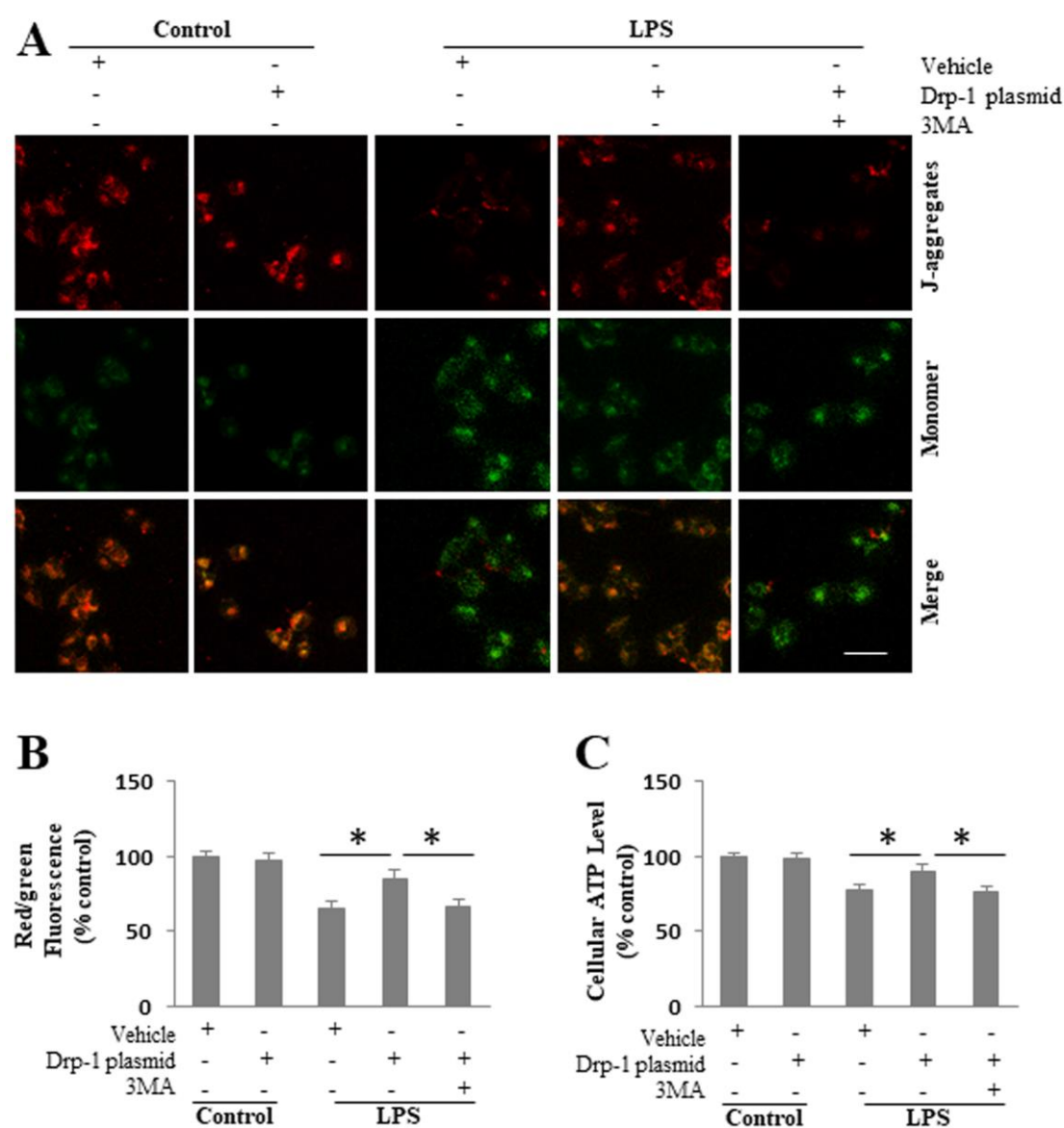

Supplement: Supplementary Materials — Supplementary Figure S1: Drp-1 regulates mitochondrial fission, and PINK1-Parkin-mediated mitophagy PMVECs were transfected with either Drp-1 siRNA or Drp-1 plasmid and exposed to LPS (500 ng/ml) for 24 h. (a) Mitochondria were stained by MitoTracker Red and imaged by laser scanning confocal microscopy. Scale bars, 10 μm. (b) Western blotting was used to assess PINK1 and Parkin levels. (c) PINK1 level quantification; (d) cytoplasmic Parkin level quantification; (e) mitochondrial Parkin level quantification. The mean ± SD represent the data (n = 6 in each group). An asterisk (∗) indicates P < 0.05 vs. the various groups. Drp-1: Dynamin-related protein-1; LPS: lipopolysaccharide; PINK1: tensin homolog (PTEN)-induced putative kinase 1; Cyto.: cytoplasmic; Mito.: mitochondrial; COX IV: cytochrome C oxidase subunit 4. Supplementary Figure S2: PMVECs transfected with Drp-1 plasmid (a null-plasmid was used as the control) were exposed to LPS (500 ng/ml) and treated with 3MA (5 mM) or a vehicle for 24 h. (a) Fluorescent inverted microscopy (200x magnification) determination of JC-1 intracellular green and red fluorescence. Scale bars, 50 μm. (b) JC-1 emitted intracellular green and red fluorescence quantification. (c) Luciferase-based assay for intracellular ATP levels. The mean ± SD represent the data (n = 6 in each group). An asterisk (∗) indicates P < 0.05 vs. the various groups. Drp-1: Dynamin-related protein-1; LPS: lipopolysaccharide. [file 5820245.f1.pdf]
